# Fish and fishery historical data since the 19th century in the Adriatic Sea, Mediterranean

**DOI:** 10.1038/sdata.2017.104

**Published:** 2017-09-12

**Authors:** Tomaso Fortibuoni, Simone Libralato, Enrico Arneri, Otello Giovanardi, Cosimo Solidoro, Saša Raicevich

**Affiliations:** 1 National Institute of Oceanography and Experimental Geophysics (OGS), Division of Oceanography, ECHO Group, Trieste, Italy; 2 Italian National Institute of Environmental Protection and Research (ISPRA), Chioggia, Italy; 3 AdriaMed Project, FAO Fisheries and Aquaculture Department, Rome, Italy; 4 Institute of Marine Sciences, National Research Council (CNR-ISMAR), Ancona, Italy; 5 The Abdus Salam International Centre for Theoretical Physics, Trieste, Italy

**Keywords:** Marine biology, Biodiversity, Community ecology

## Abstract

Historic data on biodiversity provide the context for present observations and allow studying long-term changes in marine populations. Here we present multiple datasets on fish and fisheries of the Adriatic Sea covering the last two centuries encompassing from qualitative observations to standardised scientific monitoring. The datasets consist of three groups: (1) early naturalists’ descriptions of fish fauna, including information (e.g., presence, perceived abundance, size) on 255 fish species for the period 1818–1936; (2) historical landings from major Northern Adriatic fish markets (Venice, Trieste, Rijeka) for the period 1902–1968, Italian official landings for the Northern and Central Adriatic (1953–2012) and landings from the Lagoon of Venice (1945–2001); (3) trawl-survey data from seven surveys spanning the period 1948–1991 and including Catch per Unit of Effort data (kgh^−1^ and/or nh^−1^) for 956 hauls performed at 301 stations. The integration of these datasets has already demonstrated to be useful to analyse historical marine community changes over time, and its availability through open-source data portal will facilitate analyses in the framework of marine historical ecology.

## Background & Summary

In the last two decades, the interest in the recovery, digitisation and analysis of historical data on fish and fisheries has greatly increased in the framework of marine historical ecology (MHE). At present, MHE has contributed significantly to the understanding of past conditions of the marine environment around the world, when the human impact was different from today, and in some cases, MHE has also informed management and policy in a concrete and operational way^1^.

Historical evidence provides the context for present-day observations and allows studying long-term changes in marine populations and ecosystems. Intergenerational amnesia about the composition and abundance of marine species can lead to shifting baselines^2^, the incremental lowering of ecological standards^3^, with potential consequences on current species and ecosystems management. Historical information included in marine population assessments revealed larger declines compared to those detected with short-term observations alone^4,5^. Historical data may also be used to set reference points and recovery targets in marine resource management^4^, as well as to assess the vulnerability of exploited species^1^.

Time‐series of modern scientific surveys and fishery data collection programmes in the Mediterranean (including the Adriatic Sea) span at maximum a couple of decades. Indeed, the MEDiterranean International Trawl Survey (MEDITS) programme, the only systematic survey of the demersal communities of the entire Adriatic that is still ongoing, was established in 1994 in Italy and in 1996 in Albania, Croatia, and Slovenia, whilst Montenegro joined the survey in 2008. Additionally, since 2000 an EU framework for the collection, management and use of fisheries data is in place that was reformed in 2008 resulting in the Data Collection Framework^6^. In this context, the EU Member States collect a wide range of fisheries data needed for scientific advice, landings and scientific surveys included. However, given the shortness of these standardised time series, it is difficult to resolve the relative impacts of different human and natural drivers on long‐term dynamics of marine populations. Thus, there is need to broaden ecological timelines using historical sources^7^.

Historical data collection is time-consuming and difficult, being records reported in different languages, only accessible in small archives/libraries for which no electronic resources exist, or buried in documents created for a different purpose^4^. Recovery of historical data, and efforts to make them accessible should facilitate their use in academic and management contexts.

An estimated 99% of ecological data remains inaccessible after publication^8^, while data created using public funds or for the public good should be publicly available^9^. These considerations seem to be even more drastic for the Adriatic and the Mediterranean Sea. European Commission Marine Knowledge 2020 initiative is focused on centralising marine data from different sources through the European Marine Observation and Data Network (EMODnet). Many policy instruments, indeed, require time-series and historical trends for their implementation, e.g., the European Union Common Fisheries Policy^10^ and the EU Marine Strategy Framework Directive^11^.

The lack of long-time series on Adriatic marine populations, the difficulty in accessing available fishery data, as well as the long history of interactions between humans and the environment, have motivated the compilation of these historical datasets on fish and fisheries of the Adriatic Sea. They cover two centuries (1818–2012) and range from qualitative observations (naturalists’ traditional ecological knowledge) to standardised scientific monitoring (scientific ecological knowledge) of marine communities, i.e., (1) early naturalists’ accounts; (2) historical landings; (3) scientific surveys and monitoring.

Although a first initiative to make public landings for the area exist^12^, to our knowledge this is the first attempt in the Mediterranean to collect, archive and make digitally available historical marine community statistics covering such a long period of time and including data from different sources. The objective of the data collection was to identify, describe, perform the quality control, and integrate historical data for the Adriatic Sea into standardised datasets, and to make them freely available to end-users.

The analysis and integration of the datasets described in this paper have already demonstrated to be useful to reconstruct Adriatic marine community long-term changes^13–17^, and their availability through open-source data portal is going to increase analyses^8^ in the framework of MHE. Nevertheless, users are warned about the inherent limitations and differences among datasets and a careful evaluation is recommended before any analysis in order to avoid possible misuse.

## Methods

A thorough—although not exhaustive—search was conducted in local archives, libraries and museums in Venice, Padua, Rome, Trieste, Chioggia (Italy) and Split (Croatia) to collect information on Adriatic marine species from the beginning of the 19th century onwards. All the data were retrieved from scientific literature, bulletins, theses, record books and publications. The development of datasets included digitisation (from paper copies), quality control of the digitisation process, taxonomic and geographic standardisation, data integration and graphical analysis.

### Early naturalists’ accounts

Early naturalists’ accounts are an important source of past information on marine species and communities^13,14,18,19^. These documents, particularly abundant in the 19th century because of the ascendancy of the Linnaean system, provide the earlier available systematic description of species that can be used to set a historical baseline of marine biodiversity in the Adriatic Sea^14^. The dataset (Data Citation 1) includes information on fish species reported in 29 books written by 24 naturalists and published between 1818 and 1936 (Table 1). Original books were written in Italian, German or English, and species information was translated into English. Information on invertebrates and mammals was not available in most books and thus it was not included in the dataset. Naturalists’ knowledge of fish fauna was primarily based on direct observations at fish markets and at ports, interviews with fishermen, literature and on the analysis of natural history museum collections^13^. On the basis of such sources, naturalists described 255 fish species in terms of presence, perceived-abundance, habitat, distribution, seasonality, reproduction, fishery, maximum length and maximum weight (4,027 records). In some documents, maximum length was expressed in Roman *uncia* (inch) or *pes* (foot), while maximum weight in Roman pound *libra* or ounce. The lengths were transformed in cm and approximated to the half millimetre according to the following: 1 Roman inch=2.47 cm, 1 Roman foot=29.65 cm. Weight was transformed in kg according to the following: 1 pound=0.33 kg and 1 ounce=0.027 kg. In the original documents, species were cited with their old scientific names (reported in the dataset under the field ‘SYNONYM’) that were updated according to the modern nomenclature referring to the World Register of Marine Species (WoRMS) Data Citation 2. The local vernacular name of each species is also reported in the dataset (under the field ‘VERNACULAR_ITALIAN_NAME’) as mentioned by the authors. For each book, the place where the author based his work is cited, when available (under the field ‘PLACE’).

### Historical landings

In Italy, centralised reporting on landings of marine fisheries started later than other European states^20^, i.e., in 1947, by the Italian National Institute of Statistics (ISTAT). However, it is only since 1953 that landings are reported at the species level. Before that year, for some fish markets landings were reported in fragmented and disperse data sources, for instance, local statistical bulletins or fish markets’ registers. Thus, we scoured libraries, archives and fish markets to recover and digitise available landings data for the Adriatic Sea from the beginning of the 20th century. Data were digitised from the earliest year of consistent and detailed time-series (1902).

**Figure 1:**
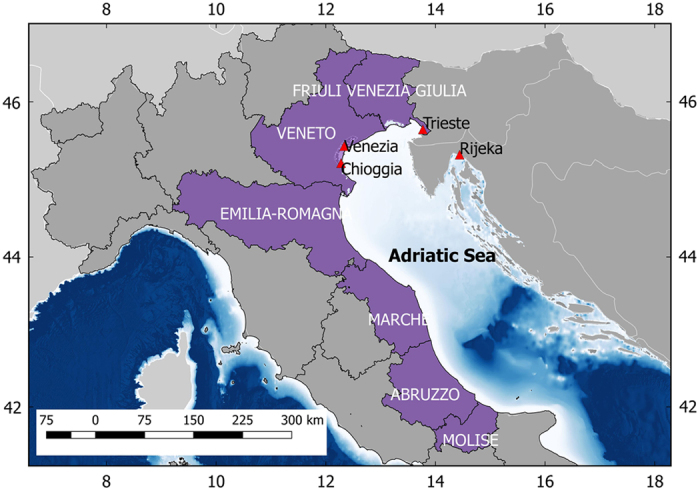
Location of the Adriatic fish markets for which historical landings are available (1902–1968) and administrative regions for which Italian official landings are available (1953–2012).

In the datasets, landings statistics from historically most important fish markets of the Northern Adriatic Sea (Venice, Chioggia, Trieste and Rijeka; Fig. 1) and wide coastal areas (Italian administrative regions: Friuli Venezia Giulia, Veneto, Emilia Romagna, Marche, Abruzzo and Molise; Fig. 1) are reported for 275 market categories in terms of annual total wet weight (kg per year) for the period 1902–2012 (Table 2). Market categories (hereafter called ‘categories’ for simplicity) vary among datasets and year according to the registration procedures adopted at the fish market in a specific year. They may include one or more species. Multispecies categories usually group taxonomically similar species, i.e., species of the same genus or family. Species were reported with their scientific names that were updated according to the modern nomenclature referring to WoRMS.

**Figure 2:**
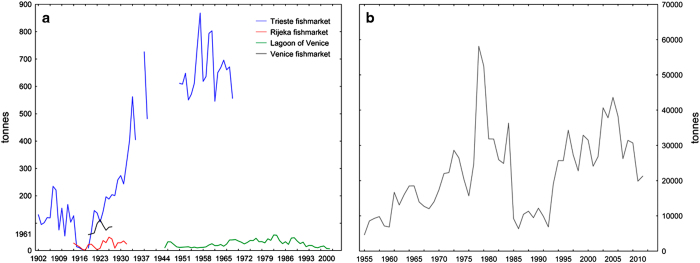
An example of landings by species. Data are referred to European anchovy (*Engraulis encrasicolus*). (**a**) Data from the Rijeka, Trieste and Venice fish markets, and from the lagoon of Venice; (**b**) Italian annual landings for the Northern and Central Adriatic Sea.

Historical landings from the Rijeka (currently Croatia) fish market for the period 1914–1932 were digitised from D’Ancona^21,22^ (Data Citation 3). At that time, the Rijeka fish market was one of the most important in the area together with Trieste and Venice. Almost all fish caught in the Northern Adriatic Sea was sold in these fish markets. The dataset contains landings for 95 categories (1,805 records). Data are referred to local capture sea-fisheries (Fig. 2a).

Historical landings from the Trieste (currently Italy) fish market for the period 1902–1968 (with some temporal gaps, see Table 2) were retrieved and entered in a digital format (Data Citation 4) from the official statistical bulletin (paper format) of the Municipality of Trieste. The dataset contains information on 177 categories (6,790 records). However, the taxonomic resolution of landings changed over time (Table 2), from a minimum number of 107 (1902) to a maximum of 140 categories (1920). Data are referred to the local consumption originated from local capture sea- and freshwater-fisheries (Fig. 2a).

Historical landings from the Venice (Italy) fish market for the period 1905–1927 (with some temporal gaps, see Table 2) were retrieved and transformed into workable spreadsheets (Data Citation 5) from statistical bulletins published by the Municipality of Venice, the ‘*Società regionale veneta per la pesca e l'acquicultura*’ (Veneto regional society for fisheries and aquaculture) and by the historical scientific journal called ‘*Neptunia*’. Landings are reported by their origin, i.e., capture-based aquaculture in the lagoon of Venice^23^, capture freshwater-fishery (local rivers and lakes), Venice lagoon capture fishery and Northern Adriatic sea capture fishery (Fig. 2a). Overall, information on 130 categories is reported (1,192 records). However, the taxonomic resolution of landings changed over time (Table 2), from a minimum number of 26 (1910) to a maximum of 108 categories (1919–1924).

The dataset (Data Citation 6) including landings for the lagoon of Venice (1945–2001) was obtained from the original registers of the fish markets of Venice and Chioggia that were available on paper documents from 1945 to 1996, and in electronic spreadsheets from 1997. The data originally included information on landings from aquaculture and wild captures from the sea- and lagoon-fisheries merged together. Thus, in order to obtain reliable time series for the lagoon-fishery alone, the raw data were treated with the aim of separating aquaculture products from sea-fisheries landings (Fig. 2a). Moreover, different unofficial sources were used to verify and correct official statistics taking into consideration unreported catch from illegal fishing^17^. Species were reported in the original documents with their vernacular/dialectal names, thus it was necessary to attribute them their scientific name according to scientific experts’ knowledge and interviews with fishermen and fish sellers. The Mediterranean shore crab (*Carcinus aestuarii*) was registered into three categories, according to the life stage: ripe females, moulting individuals, and the other specimens. The different stages have different commercial values, with moulting individuals being the most valuable^12^. The European seabass (*Dicentrarchus labrax*) was registered in two categories according to the size (juvenile or adult). Landings for 19 categories were obtained (Table 2; 1,083 records). The Chioggia fish market data are also included in the Clodia database^12^ (freely available online at http://chioggia.scienze.unipd.it/DB/database_landing.html?menu=00) where, however, landings from the lagoon and sea are not separated.

Italian annual landings (1953–2012) for the Northern and Central Adriatic Sea originated from official Italian statistics on the fishery, reported by ISTAT from 1953 to 2004, and by the Institute for Economic Research in Fishery and Aquaculture (IREPA) from 2005 to 2012 (Data Citation 7). Until 2001, landings were published in the ISTAT annual bulletins (printed format) while after 2002 they were available in digital format. The method of landings data collection changed in 2005. ISTAT collected monthly landings data (census survey) at Marine Compartment level from Port Authorities using standardised paper templates, while IREPA performed sample surveys applying the Computer-assisted personal interviewing (CAPI) technique following ISTAT guidelines. In the dataset, landings are aggregated at the level of Italian administrative regions (Fig. 1; Table 2) and do not include discarded, illegal and unreported catches. In the original documents, species were reported according to their common names, thus it was necessary to attribute them their scientific name. Common names reported in the original documents changed over time, and they were updated according to the actual official name as defined by the Italian Ministry of Agricultural, Food and Forestry Policies in 2008. Overall, information on 120 categories are available in the dataset (15,077 records), but the taxonomic resolution of landings changed over time (Table 2) ranging from a minimum of 31 (1958) to a maximum of 81 categories (1955–1957). Data refer to landings from capture sea- and freshwater-fisheries in the Adriatic Sea landed by Italian boats (Fig. 2b).

### Scientific surveys

The datasets include historical trawl-survey data from seven surveys performed with bottom otter-trawl nets and spanning the period 1948–1991. Since raw data were not accessible or publicly available, the datasets include data retrieved from scientific publications. In Table 3 the source from which data were extracted is reported for each dataset.

Overall, Catch per Unit of Effort (CPUE; kgh^−1^ and/or nh^−1^) by species for 956 hauls performed at 301 stations is reported (Table 3). When stations coordinates were not explicitly available, they were geolocated based on sampling locations as reported on printed maps included in the data source by using a GIS software. The depth of each station was extracted from the EMODnet Digital Bathymetry (Data Citation 8). The taxonomic resolution varies among datasets. Some species are grouped into multispecies categories (hereafter called ‘species’ for simplicity) that usually group taxonomically similar species, i.e., species of the same genus or family. Species synonymies were updated according to the modern nomenclature referring to WoRMS. Each dataset regards trawl-survey programs standardised in terms of average duration of the hauls, speed, gear and net mesh used (Table 4). Different standardisations of these surveys do not allow straightforward comparisons between these datasets and/or with modern long-term scientific monitoring programs (e.g., MEDITS).

**Figure 3:**
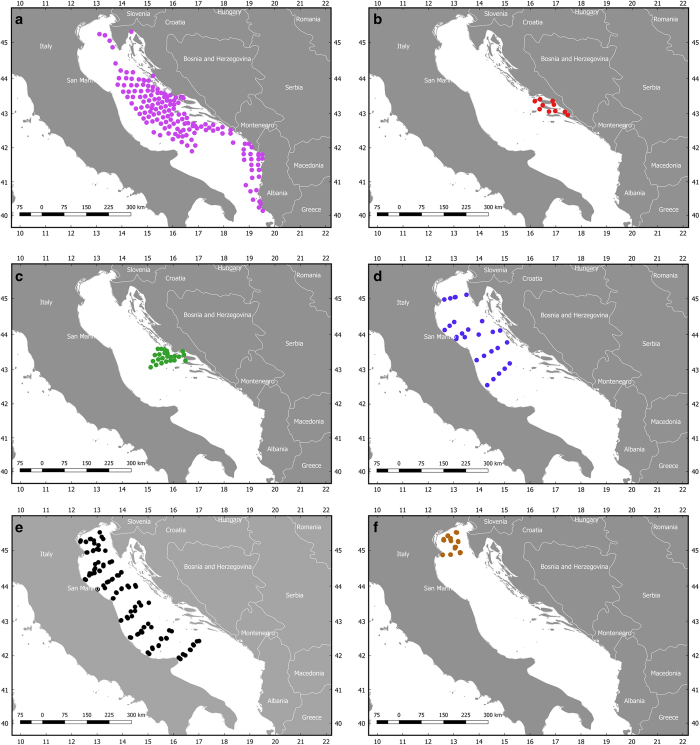
Positions of the trawl-surveys sampling stations. (**a**) Expedition HVAR (1948–1949); (**b**) Bios-Predvodnik 1 (1957–1958); (**c**) Bios-Predvodnik 2 (1956–1971); (**d**) Santi Medici (1972) and Giannetto (1975, 1981); (**e**) Pipeta 1 (1982); (**f**) Pipeta 2 (1988, 1991).

The ‘expedition HVAR’ (1948–1949), organised by the Institute of Oceanography and Fisheries of Split (IOF, Croatia) between January 8th 1948 and 31st March 1949, was the first large-scale fishery-independent trawl-survey ever performed in the Adriatic Sea. The survey was conducted with the research vessel ‘Hvar’ (250 HP; LOA=25 m) in the territorial waters of the former Yugoslavia (currently territorial waters of the Republics of Slovenia, Croatia, Bosnia and Herzegovina and Montenegro) and Albania, and in the international waters to approximately 20 nautical miles off the Italian coast (Fig. 3a). The dataset (Data Citation 9) includes CPUE (nh^−1^ and kgh^−1^) indexes of 95 demersal species for 271 hauls at 151 fixed stations (Table 3; 25,745 records). Information on the sediment type was also reported for each station. The trawling was carried out during daylight. The purpose of the research was to determine the qualitative and quantitative characteristics of the demersal communities of fish, crustaceans and cephalopods, and to assess the possibility of their exploitation^24^. This survey was conducted in the period when the demersal fish and invertebrate communities were not intensely exploited (‘post-war conditions’^25^), and thus this data can serve as a reference baseline for the following changes^24^.

Between June 5th 1957 and July 4th 1958 a series of trawl-surveys (hereafter referred as Bios-Predvodnik 1) were organised by IOF in the Croatian channels around the Hvar Island (Central-eastern Adriatic, Fig. 3b). Tows were performed once a month in all of the 10 fixed stations, for a total of 120 hauls (Table 3). Two research vessels were employed, ‘Bios’ (300 HP; LOA=26 m) and ‘Predvodnik’ (200 HP; LOA=19 m). CPUE (nh^−1^) indexes for 104 demersal species were recorded (6,718 records), as well as information on the sediment type at each station (Data Citation 10). Sediment samples were taken by means of the Peterson power shovel.

Between October 1956 and January 1971, IOF performed another series of trawl-surveys (hereafter referred as Bios-Predvodnik 2) in the Croatian coastal waters between Split and Šibenik (Central-eastern Adriatic, Mediterranean, Fig. 3c) in the area of the Jabuka Pit with the above-mentioned boats (Bios and Predvodnik). The samples were collected at some stations of the ‘expedition Hvar’. 447 hauls were carried out at 26 fixed stations (Data Citation 11). CPUE (nh^−1^ and kgh^−1^) indexes were derived for 129 demersal species (19,756 records). Information on the sediment type at each station was also recorded using an echo sounder (Table 3).

In November 1972, the Laboratory of Marine Biology and Fisheries of Fano (LMBF, Italy) and IOF organised a fishery-independent joint research in the Central Adriatic along the profile Fano-Dugi Otok (5 hauls), which was extended to four profiles (17 hauls) in the Northern and Central Adriatic in October 1975. In September 1981, another survey performed three profiles in the Northern Adriatic Sea (9 hauls), replicating some of the stations sampled in 1975 (Fig. 3d; Table 3). Demersal assemblages (41 species in 1972, 59 species in 1975 and 36 in 1981) were sampled with the use of a bottom otter-trawl net and reported in terms of quantity and quality (CPUE: kgh^−1^). Sampling in 1972 was carried out by means of the Italian commercial trawler ‘Santi Medici’ (300 HP; LOA=unknown), while in 1975 and 1981 by means of the Italian commercial trawler ‘Giannetto’ (120 HP; LOA=19 m). Some information is available on the characteristics of the nets employed during these surveys (Table 4). These three surveys were included in the same dataset (1,471 records) (Data Citation 12).

The ‘Pipeta programme’ (named after the Italian commercial trawler used; 300 HP; LOA=26 m) was started in 1982 by LMBF and IOF. The expedition was a fishery-independent trawl-survey of the demersal communities using a bottom otter-trawl net (Table 4). The survey was successively named GRUND programme and ended in 2007.

The first ‘Pipeta’ dataset (hereafter referred as Pipeta 1; Data Citation 13) includes CPUE (nh^−1^ and kgh^−1^) indexes for 187 benthic invertebrate species recorded between May 3rd 1982 (spring survey) and December 16th 1982 (autumn survey) at 61 stations (Fig. 3e; Table 3). Information on bottom type was also reported at each station. The weight of invertebrate catch was calculated as follows: a plastic fish-box of dimensions 50×32×10.5 cm was filled with a random sub-sample of the epifauna collected in the haul. The sub-sample was analysed. Then, the weight of the sub-sample was multiplied by the total number of fish-boxes collected in each haul. Large-sized specimens (e.g., *Atrina pectinata*, *Gracilechinus acutus*) were separated, counted and weighted first. Three replicates of each haul were performed during spring survey, while during autumn two replicates of each haul were performed. In the dataset, the mean value of coordinates and CPUE are reported for each station (2,451 records).

The second ‘Pipeta’ dataset (hereafter referred as Pipeta 2; Data Citation 14) includes a subset of 26 stations (Northern Adriatic Sea) for the years 1988 (January and October) and 1991 (January) from the ‘Pipeta programme’ (Fig. 3f). Two successive trawl hauls were performed at each station, with the second tow frequently performed along the same distance but in the opposite direction. Data per stations are given as the mean value of two replicates. CPUE (kgh^−1^) indexes for 166 demersal and benthic species (1,807 records) and information on sediment type are reported (Table 3). The data included in the dataset was used to analyse the effect of anoxia/hypoxia events on benthic and demersal communities. In October 1988, hypoxic state (oxygen concentrations between 0.97 and 1.88 ml l^−1^) was observed at stations A4b, A7c and B3c, and at stations A4b and B2c mass mortality of benthic organisms occurred.

## Data Records

The 14 datasets are available in EMODnet Biology (http://www.emodnet-biology.eu/) under Creative Commons Attribution 4.0 International Public License. EMODnet Biology assembles individual datasets from various sources and processes them into interoperable data products implementing common standards defined by SeaDataNet, WoRMS, OBIS (Ocean Biogeographic Information System), INSPIRE, GBIF (Global Biodiversity Information Facility) and the Lifewatch infrastructure. EMODnet Biology hosts also datasets recovered from personal files and documents that would otherwise be lost or inaccessible, like the datasets described in this paper.

## Technical Validation

### Early naturalists’ accounts

Naturalists’ books from which information on Adriatic fish species was retrieved originally included information on 394 fish species. Species’ list was checked for accuracy and 139 species were excluded from the dataset, i.e., species cited by fewer than five authors, species belonging to freshwater habitats, species that were misreported (e.g., the Atlantic cod *Gadus morhua*), and misspelt species (e.g., *Laeviraja morula* and *Notidanus barbarus*)^13^. In order to minimise the possibility that naturalists were copying each other, we checked in each book cross references and included in this study only documents primarily based on new information gathered directly by each author. Naturalists recorded the presence of species but not typically their absence, such that one cannot be sure whether a particular species was not present in the past or present but not recorded. Conversely, species perceived abundance, when reported by naturalists, can provide potential information on changes through time of species abundance at sea. The perceived abundance of the species was ranked using a four-level class coding system (i.e., very rare, rare, common and very common)^13^. The dataset was analysed by Fortibuoni *et al.*^13^ to reconstruct and quantitatively analyse a 200-year-long time series of fish community structure indicators in the Northern Adriatic Sea. A methodology allowing the coding of qualitative information provided by naturalists into semi-quantitative information through an intercalibration with landings proportions was defined. Long-term changes in fish community structure, including the decline of Chondrichthyes, large-sized and late-maturing species, were described. A further analysis on the dataset allowed defining the historical baseline for Common Angelshark (*Squatina squatina*) in the Northern Adriatic Sea^14^. According to naturalists’ accounts, in the 19th and early-20th centuries, the species was so abundant in the area that it sustained targeted fisheries.

### Historical landings

Temporal changes in landings in terms of relative abundance of species or groups of species can be used as a proxy for studying fish community structure changes over time^12,26^. An analysis of the relative composition of historical landings from the Northern Adriatic Sea included in the present datasets (Rijeka, Trieste and Venice fish markets) was first done in the early 20th century by the Italian biologist Umberto D’Ancona^21^. The author described the increase in predator fish and a decrease in prey fish of various species in the Adriatic Sea during the World War I period (WWI). From a historical ecology perspective, the WWI represented a large-scale ecological experiment, since it caused the release of the main anthropogenic pressure affecting the Adriatic ecosystem (i.e., fishing) that allowed predator species to recover. Using the same data, the Italian mathematician Vito Volterra developed the first and simplest model of predator–prey interactions, later known as Lotka-Volterra equations^27^. Historical landings data included in the datasets were also analysed by Fortibuoni *et al.*^13^ and integrated with naturalists’ descriptions of fish fauna to reconstruct long-term changes in fish community structure (see the previous paragraph). Landings from the lagoon of Venice (1945–2001) were validated and analyzed by Libralato *et al.*^17^ to describe the long-term modifications in the lagoon ecosystem structure, to identify the major forces which had been driving its dynamics, and to evaluate the effects of the introduction and the mechanical exploitation of the non-native species Manila clam (*Ruditapes philippinarum*). Four ecological stages similar to those considered typical of exploited marine systems subjected to nutrient enrichment^28^ were identified. A subset (1972–2012) of the Adriatic landings dataset was included in the analysis aimed at testing if an increase of warmer-water species against colder-water ones was observed in Italian fisheries^16^. The Mean Temperature of the Catch (MTC)^29^ was computed for different basins. Interestingly, while global MTC increased at a rate of 0.12 °C per decade, in the Northern Adriatic Sea a decrease of 0.14 °C was observed and an inverse relationship was found between MTC and sea surface temperature (SST). To overcome problems related to changes in the taxonomic resolution of landings over time, only species clearly recognisable across the entire time-series were included in the analysis. The final dataset resulted to be composed of 25 species in each region.

### Scientific surveys

Historical trawl-surveys data included in these datasets (i.e., HVAR, Bios-Predvodnik 1 and 2) were compared with recent trawl-surveys data by Ferretti *et al.*^15^ to examine spatial and temporal changes in the elasmobranch community of the Adriatic Sea. Long-term community changes were estimated by comparing catches across surveys. The authors found that the high elasmobranch abundance and diversity characterising the Central Adriatic during the ‘expedition Hvar’ disappeared, and detected a structurally depleted elasmobranch community. Fishing was a major driver of change, while differences in the intrinsic vulnerabilities among species did not allow explanation of species-specific rates of change. A previous work^25^ compared ‘expedition HVAR’ data with Mediterranean international trawl survey (MEDITS) programme data for 1998 to detect long-term changes in demersal resources. The main change observed was the decrease of elasmobranchs diversity and occurrence.

## Usage Notes

Previous analyses highlighted the potential usefulness of this data for unravelling the long-term changes of the Adriatic marine communities, through the integration of different sources^13,14^, application of indices^16,17^ or complex statistical analysis^15^. However, in all these applications particular care was used to overcome the limitations of the datasets and to find approaches that allowed their appropriate use. It is worth noting that during the period covered by the datasets (19th and 20th centuries), dramatic geopolitical changes occurred in the area. Such changes may have influenced the activities of fishermen and naturalists, in particular as regards the geographical scope of landings and naturalists’ data. It is the responsibility of investigators to understand the limitations of the data and apply it appropriately^8^.

Data from these datasets may be compared to long-term environmental data (e.g., oxygen content^30^, nutrient load^31^, water transparency^32^, eutrophication^33,34^, anoxic events^35^) and other historical information (e.g., benthic fauna^36^, benthic algal flora^37^) available for the Adriatic possibly allowing to disentangle the role of different drivers in shaping fish communities. However, users should take into account that these datasets do not contain absolute biomass at sea for the species, thus some comparisons might be meaningless.

For the naturalists’ dataset, it was not possible to completely eliminate the possibility that the authors were copying each other, and thus some information reported in different documents may be not independent. As regards books written by the same author, information reported cannot be considered independent. The city where naturalists based their work is reported in the dataset, however, apart from some cases (see field ‘DISTRIBUTION’), the spatial scale of observations is not mentioned in the books and thus the geographical scope of the dataset was generally defined as the Adriatic Sea.

Concerning landings, the intrinsic limitations of fishery-dependent data hamper the possibility of deriving biological densities directly from catch statistics^38,39^. Indeed, rather than only changes in abundance at sea, the temporal changes in landings may be due also to changes in fishing techniques, fishing equipment, fishermen behaviour, changes in market demand, the introduction of regulations and laws, management and economic factors, etc. that might need to be considered before analysis. Moreover, while for species with a high commercial value the discarded fraction of the catches is usually low, for low-value species the discarded quantities can be large. Thus, landings may underestimate the catches for these resources. Landings data do not indicate the physical location of harvest (i.e., the geographical area exploited) but the location at which catches were landed. Finally, data from different datasets can hardly be integrated into consistent time-series at the species level, since the reporting method, taxonomical resolution and the reliability of the data may have changed through time and among sources. In some instances, this problem may be overcome by aggregating species to a coarser taxonomic level (e.g., functional groups^26^).

When comparing CPUE from different trawl-surveys, caution is recommended since different vessels, gears and protocols were used in different surveys (Table 4). Consequently, even if available gear information and speed allowed estimating the swept area in some cases^40–42^, the catching efficiency and the selectivity may broadly differ among surveys. Moreover, the use of this data for community studies is strongly discouraged not only because of differences in catchability but also because during surveys, not all caught species have been recorded or reported and this varies depending on the scope.

## Additional Information

**How to cite this article**: Fortibuoni, T. *et al.* Fish and fishery historical data since the 19th century in the Adriatic Sea, Mediterranean. *Sci. Data* 4:170104 doi: 10.1038/sdata.2017.104 (2017).

**Publisher**’**s note**: Springer Nature remains neutral with regard to jurisdictional claims in published maps and institutional affiliations.

## Supplementary Material



## Figures and Tables

**Table 1:** List of naturalists’ book included in the dataset.

**Year**	**Author**	**Book title**	**Place**	**Species**
1818	S. Chiereghin	Descrizione de' pesci, de' crostacei, e de' testacei che abitano le lagune ed il golfo veneto	Chioggia	120
1822	F.L. Naccari	Ittiologia adriatica	Chioggia	89
1823	G.D. Nardo	Descrizione di un pesce raro dell'Adriatico ed osservazioni ittiologiche dedicate al signor Giuseppe Cernazai	Chioggia	119
1824	G.V. Martens	Riese nach Venedig	Venice	111
1827	G.D. Nardo	Prodromus observationum et disquisitionum adriaticae ichthyologiae	Chioggia	129
1832-1841	C.L. Bonaparte	Iconografia della fauna italica per le quattro classi degli animali vertebrati - tomo III (pesci)	NA	54
1846	E. Plucar	Der fischplatz zu Triest oder aufzählung und populäre beschreibung der demselben aus dem Adriatischen Golfe zugeführten fische	Trieste	109
1847	G.D. Nardo	Prospetto della fauna marina volgare del veneto estuario con cenni sulle principali specie commestibili dell'Adriatico	Chioggia	36
1860	G.D. Nardo	Prospetti sistematici degli animali delle province venete e del mare Adriatico e distinzione delle specie in gruppi relativi alla loro geografica fisica ed all'interesse economico statistico che presentano	Venice	219
1870	A.P. Ninni	Enumerazione dei pesci delle lagune e golfo di Venezia	Venice	219
1872	A. Targioni-Tozzetti	La pesca in Italia. vol. I, pt II	NA	212
1875	E.F. Trois	Prospetto sistematico dei pesci dell'Adriatico e catalogo della collezione ittiologica del Regio Istituto Veneto	Venice/Trieste	205
1876	S. De Syrski	Relazione sulle osservazioni fatte riguardo al tempo della frega degli animali esistenti nel mare Adriatico	Trieste	96
1879	M. Stossich	Prospetto della fauna del mare Adriatico	Trieste	227
1879	P. Doderlein	Manuale ittiologico del Mediterraneo	NA	46
1880	A.P. Ninni	La pesca nei mari d'Italia e la pesca all'estero esercitata dagli italiani	Venice	97
1880	E.H. Giglioli	Elenco dei mammiferi, degli uccelli e dei rettili ittiofagi appartenenti alla fauna italica: e catalogo degli anfibi e dei pesci italiani	NA	161
1880	L. Sormani-Moretti	La Provincia di Venezia: monografia statistica, economica, amministrativa - fauna	Venice	240
1881	A. Perugia	Elenco dei pesci dell'Adriatico	Trieste	208
1881	J. Kolombatovic	Mammiferi, anfibi e rettili, e pesci rari e nuovi per l'Adriatico catturati nelle acque di Spalato	Split	61
1882	C. De Marchesetti	La pesca lungo le coste orientali dell'Adria	Trieste	177
1883	G.L. Faber	The fisheries of the Adriatic and the fish thereof	Rijeka	252
1895	V.L. Sucker	Die fische nebst den essbaren wirbellosen thieren der Adria und ihre zubereitung	Trieste	177
1912	E. Ninni	Catalogo dei pesci del mare Adriatico	Venice	206
1913	G. Pastrovic	Manuale del pescatore per l'anno 1913	Trieste	70
1917	E. Ninni	La pesca nel mare Adriatico	Venice	69
1920	E. Ninni	Pesci, crostacei e molluschi nel vernacolo veneziano	Venice	132
1928	A. Vatova	Compendio della flora e fauna del mare Adriatico presso Rovigno	Rovinj	122
1936	R. Cella	Il pescatore dilettante - lo sport della pesca in Alto Adriatico	Rijeka	64
The place where the author based his work is cited as well as the number of species described.				

**Table 2:** List of historical landings datasets.

**Market/Region**	**Years**	**Species**	**Origin**
Rijeka	1914–1932	95	Sea-fishery
Trieste	1902–1917; 1919–1935; 1938–1939; 1950–1968	177 (107–140)	Sea-fishery; freshwater-fishery
Venezia	1905; 1910; 1919–1927	130 (26–108)	Sea-fishery; freshwater-fishery; lagoon-fishery; lagoon-aquaculture
Chioggia/Venezia	1945–2001	19	Lagoon-fishery
Northern and Central Adriatic Sea	1953–1972; 1974–2012	120 (31–81)	Sea-fishery; freshwater-fishery
The total (and min-max range in brackets) number of species is reported, as well as the origin of the product.			

**Table 3:** List of trawl-surveys datasets.

**Survey**	**Years**	**Stations**	**Hauls**	**Depth range (m)**	**Species**	**Taxonomic scope**	**CPUE**	**Additional parameters**	**Source**
HVAR	1948–1949	151	271	7–566	95	Demersal fish	nh^−1^; kgh^−1^	Sediment type	Karlovac, O. (1959). Istraživanja naselja riba i jestivih beskralježnjaka vučom u otvorenom Jadranu=Exploration of fish stocks and edible invertebrata carried out by trawling in open Adriatic. The M.V. ‘Hvar’ cruises researches into fisheries biology. Institut za Oceanografiju i Ribarstvo (Split)
BIOS-PREDVODNIK_1	1957–1958	10	120	26–103	104	Demersal fish	nh^−1^	Sediment type	Županović, S. (1961). Kvantitativno-kvalitativna analiza ribljih naselja kanala srednjeg Jadrana=Analyse quantitative-qualitative des populations des poissons dans les canaux de l’Adriatique moyenne. Acta Adriatica, 9(3): 1–151
BIOS-PREDVODNIK_2	1956–1971	26	447	34–230	129	Demersal fish	nh^−1^; kgh^−1^	Sediment type	Jukić, S. (1975). Kocarska podrucja u srednjem Jadranu=Trawl fishing grounds in the central Adriatic. Acta Adriatica, 17(1): 1–86; Zupanovic, S., and Jardas, I. (1989). Fauna i flora Jadrana—Jabučka Kotlina. Logos, Split
SANTI_MEDICI	1972	5	5	15–71.6	41	Demersal fish	kgh^−1^		Jukić, S., and Piccinetti, C. (1981). Quantitative and qualitative characteristics of demersal resources in the Adriatic Sea with some population dynamics estimates. General Fisheries Council for the Mediterranean (GFCM). Report of the second Technical Consultation on Stock Assessment in the Adriatic, Ancona, 18–22 May 1981
GIANNETTO	1975; 1981	22	26	13–253	68	Demersal fish	kgh^−1^		Jukić, S., and Piccinetti, C. (1981). Quantitative and qualitative characteristics of demersal resources in the Adriatic Sea with some population dynamics estimates. General Fisheries Council for the Mediterranean (GFCM). Report of the second Technical Consultation on Stock Assessment in the Adriatic, Ancona, 18–22 May 1981; Arneri, E. (1981). Osservazioni sulle risorse demersali dell’Alto e Medio Adriatico. Master of Science Thesis (University of Bologna, Italy)
PIPETA_1	1982	61	61	11–464.4	187	Benthos	nh^−1^; kgh^−1^	Bottom type (biocenosis)	Šimunović, A. (1997). Quantitative and qualitative investigations of benthic communities in the areas of mobile bottoms of the Adriatic Sea. Acta Adriatica, 38(1): 77–194
PIPETA_2	1988; 1991	26	26	19.4–40.4	166	Demersal fish; Benthos	kgh^−1^	Sediment type	Šimunović, A., Piccinetti, C., and Zore-Armanda, M.-N. (1999). Kill of benthic organisms as a response to an anoxic state in the northern Adriatic Sea (a critical review). Acta Adriatica, 40(1): 37–64
For each dataset the source from which the data were extracted is reported. CPUE, catch per unit of effort.									

**Table 4:** Summary of vessels, gears and sampling characteristics of the trawl-survey included in the datasets.

**Survey**	**Gear**	**Tow speed (kn)**	**Tow duration (min)**	**Wing-mesh (mm)**	**Cod-end mesh (mm)**	**Foot rope (m)**	**Head rope (m)**	**Horizontal opening (m)**	**Swept area (km**^**2**^**/h)**
expedition HVAR	cotton bottom otter-trawl net	3	60 (50–120)*	55	26	44	35	NA	NA
Bios-Predvodnik 1	cotton bottom otter-trawl net	NA	60	57	22	34	28	NA	NA
Bios-Predvodnik 2	cotton bottom otter-trawl net	3	60	55	20	36.3	29.1	NA	NA
	cotton bottom otter-trawl net	3	60	55	20	46.1	34.1	NA	NA
	synthetic bottom otter-trawl net	3	60	50	20	40	37	NA	NA
Santi Medici	synthetic bottom otter-trawl net	3.5	60	55	20	NA	NA	9†	0.058338†
Giannetto	synthetic bottom otter-trawl net	3	60	NA	18	NA	NA	8‡	0.044448‡
Pipeta programme	synthetic bottom otter-trawl net	3.5	60§	55 (108 opening)	22 (40 opening)	41	32	12||	0.077784||
NA=not available.									

*Haul duration varied between 50 to 120 min (tow duration of most hauls being 60 min).

^†^Estimated by Jukic and Piccinetti^40^.

^‡^Estimated by Arneri^41^.

^§^Sampling duration was 60 min for almost all hauls, with the exception of one haul (sampling duration of 15 min).

^||^Estimated by Piccinetti *et al.*^42^.
