# Erratum: Fish and fishery historical data since the 19th century in the Adriatic Sea, Mediterranean

**DOI:** 10.1038/sdata.2018.144

**Published:** 2018-07-24

**Authors:** Tomaso Fortibuoni, Simone Libralato, Enrico Arneri, Otello Giovanardi, Cosimo Solidoro, Saša Raicevich

*Scientific Data* 4:170104 doi: 10.1038/sdata.2017.104 (2017), Published 12 September 2017; Updated 24 July 2018

In the HTML version of this Data Descriptor, Data Citation 8 incorrectly listed the repository as *VLIZ* instead of *EMODnet Bathymetry*.

